# The Role of Anger in Motivating Leadership

**DOI:** 10.3389/fmed.2021.613977

**Published:** 2021-06-25

**Authors:** Iona Heath, Anna Stavdal, Johann Agust Sigurdsson

**Affiliations:** ^1^Royal College of General Practitioners, London, United Kingdom; ^2^World Organization of Family Doctors (WONCA World), General Practitioner, Norwegian College of General Practice, Oslo, Norway; ^3^Nordic Federation of General Practice, Reykjavík, Iceland; ^4^General Practitioner, Primary Healthcare Center Grafarvogur, Reykjavík, Iceland; ^5^General Practice Research Unit, Department of Public Health and Nursing, Norwegian University of Science and Technology, Trondheim, Norway

**Keywords:** core values, leadership, general practice, family medicine, anger, hope

## Abstract

As doctors, we see every working day the pervasive effects of different forms of structural violence and discrimination that undermine the hopes and aspirations of those on the losing side. This leads to powerlessness, fear and anger. Anger is not only forward facing but also directed toward, systems, institutions, governments—rather than individuals. At its best it is a protest against the status quo. We point out that leadership is one of the core values of our professionalism. In the light of what we see and hear, we have a responsibility to use the anger that this engenders within us to speak truth to power: this speaking is leadership. Our message is: feel the fear and the anger, use it to change the world, and enfold leadership in hope and the pursuit of justice.

Most general practitioners/family physicians (GPs) work in the clinical setting, but many also take on administrative, academic, and teaching responsibilities. They are involved in teams locally, regionally, nationally, and sometimes globally. Part of their professional responsibility is to observe, analyze the problem, and take appropriate action (1). As a direct result, GPs have many exceptional opportunities to influence the evolution of healthcare. The Nordic Colleges of General Practice/Family Medicine have recently endorsed and published their *Core Values and Principles*, where they explicitly state what GPs stand for and intend to act upon (2, 3). One of the main messages is that leadership is part of the professionalism. The Nordic GPs state that they consider it their duty to take the lead. This paper will argue that some of the best leadership occurs at the intersection of anger, fear, justice, and hope.

On May 1, 2017, the philosopher Martha Nussbaum gave the Jefferson Lecture in the Humanities, which is “the highest honor the [US] federal government confers for distinguished intellectual achievement in the humanities.” Her title was “Powerlessness and the Politics of Blame,” but her subject was anger. She explored the fearfully destructive urge that leads from anger to revenge and retribution but acknowledged that:

there is one species of anger, I believe, that is free of the retributive wish: its entire content is “How outrageous that is. Something must be done about that.” I call this “Transition Anger,” because it expresses a protest, but faces forward: it gets to work finding solutions rather than dwelling on the infliction of retrospective pain.

Our view is that this Transition Anger is not only forward facing but also directed toward systems, institutions, and governments—rather than individuals. At its best, it is a protest against the status quo, for example, against the structural violence that scars and diminishes far too many lives or against corporate greed and the destruction of the nature and the environment: it is the pursuit of justice, informed by hope. It is the anger that has brought young people across the world onto the streets to protest against climate change and the anger that has motivated Black Lives Matter in response to the recurrent abuse and murders endured by Black people over all the generations since the beginnings of colonization and slavery.

As doctors, we see every working day the effects of the structural violence of socio-economic and gender inequality, racism, homophobia, and all other forms of discrimination that systematically undermines the hopes and aspirations of those on the losing side. And as a direct result of what we see and hear, we have a responsibility to use the anger that this engenders within us to speak truth to power: this speaking is leadership. We must help to seek justice for those whose health and lives are being actively damaged, always informed by the hope and the conviction that the current state of affairs is not fixed and that change is possible.

Another American writer Rebecca Solnit recognizes the power of hope:

Actions often ripple far beyond their immediate objective, and remembering this is reason to live by principle and act in hope that what you do matters, even when results are unlikely to be immediate or obvious.

What we do matters, and because we are doctors and because we cannot fail to understand the extent to which injustice curtails lives, we hold a responsibility for hopeful anger on behalf of those for whom we strive to provide care.

This responsibility comes freighted with fear, and again, as doctors, we know how closely related are fear and anger. We know that when a patient seems inappropriately angry, we should look for the hidden fear that is driving that anger. And we too can use anger to channel our fear of speaking out and of taking on this sort of leadership role.

The great Scottish writer Robert Louis Stevenson understood that anger can also be a source of the necessary courage:

that kind of anger of despair that has sometimes stood me in stead of courage.

In these ways, we discover that the key to Martha Nussbaum's transition anger is that it is felt on behalf of others rather than oneself. Nussbaum herself points out the extent to which fear for what we love triggers anger—and this happens to healthcare workers almost every day—our love for our patients and our colleagues making us fearful for them, and then angry.

So, the message is to feel the fear and the anger and use it to change the world and enfold leadership in hope and the pursuit of justice (Figure 1).

**Figure 1:**
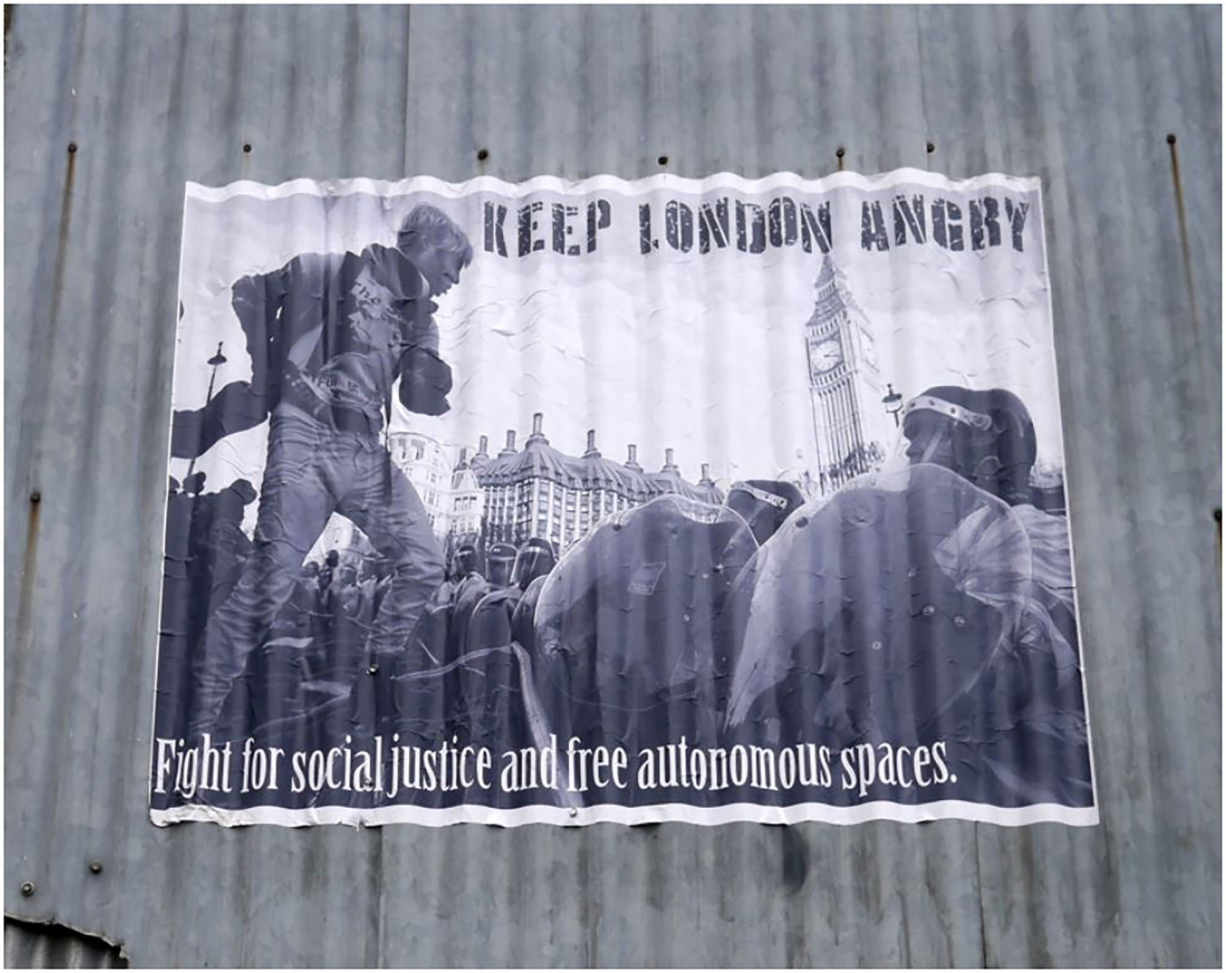
Feel the anger and use it.

## Author Contributions

IH made the first draft. All authors have contributed to the content.

## Conflict of Interest

The authors declare that the research was conducted in the absence of any commercial or financial relationships that could be construed as a potential conflict of interest.
